# Quality of life and Q-TWiST were not adversely affected in Ewing sarcoma patients treated with combined anlotinib, irinotecan, and vincristine (Peking University People's Hospital Ewing sarcoma trial-02, PKUPH-EWS-02)

**DOI:** 10.1097/MD.0000000000028078

**Published:** 2021-12-23

**Authors:** Sen Dong, Kunkun Sun, Lu Xie, Jie Xu, Xin Sun, Tingting Ren, Yi Huang, Rongli Yang, Xiaodong Tang, Fan Yang, Jin Gu, Wei Guo

**Affiliations:** a Musculoskeletal Tumor Center, Peking University People's Hospital, Beijing, 100044, China; b Pathology Department, Peking University People's Hospital, Beijing, 100044, China; c Radiologic Department, Peking University Shqougang Hospital, Beijing, 100144, China; d Surgical Oncology, Peking University Shougang Hospital, Beijing, 100144, China.

**Keywords:** advanced Ewing sarcoma, dynamic change, health-related quality of life, Q-TWiST analysis

## Abstract

**Background:**

Combined treatment with anlotinib, irinotecan, as well as vincristine for advanced Ewing sarcoma (EWS) has been verified been effective in the prospective trial of Peking University People's Hospital EWS trial-02. We aimed to assess the dynamic changes in health-related quality of life (QoL) and the benefit-risk in quality-adjusted survival in current study.

**Methods:**

Twelve “pediatric” patients and 23 “adult” patients were enrolled. QoL was assessed with the EORTC QLQ-C30 for adults and PedsQL 3.0 Cancer Module for children and adolescents. The quality-adjusted time without symptoms of disease progression or toxicity of treatment (Q-TWiST) analysis was used to describe treatment results.

**Results:**

Progression-free survival was not accompanied by diminished QoL. Differences in scores on the QoL global health status and specific functioning before, during, and after treatment were not significantly different with time (*P* = .14 for adults and .91 for children). During treatment, there was a statistically insignificant trend towards improved QoL with reduced tumor burden (*P* = .14 for adults and .10 for children), but QoL significantly declined with progression of disease (*P* = .05 for adults and .04 for children). The most common adverse events were neutropenia (12.1%), leukopenia (16.6%), anemia (12.7%), and diarrhea (4.93%). Results across the trial analyses showed that the median time of Q-TWiST was 0.73 (interquartile range, 0–1.57) months, whereas the median time with toxicity before disease progression was 3.9 (interquartile range, 2.3, 6.1).

**Conclusion:**

QoL exhibited a trend towards improvement in accordance with high objective response in this trial with the receipt of combination therapy of anlotinib, vinsristine, and irinotecan for advanced EWS. The toxicity profile did not translate into significantly worse overall scores during treatment.

## 1 Introduction

The prognosis of adolescents and young adults who have Ewing sarcoma (EWS) that is refractory to first-line chemotherapy (vincristine, doxorubicin, cyclophosphamide, ifosfamide, and etoposide, namely, VDC/IE in AWES-0031^[1]^ and EURO-Ewing 99^[2]^) remains less than 30% despite the development of systemic therapies.^[3]^ Some promising results came from a small-sample, retrospective study of 10-day irinotecan-based chemotherapy,^[4–6]^ and, as a result, an increasing number of clinicians use this combination as a second-line therapy after the failure of VDC/IE or vincristine, ifosfamide, doxorubicin, and etoposide. Although downstream targets of t(11;22)(q24;q12) translocation (EWSR1-FLI1) have been investigated through multiple trials,^[3]^ such as insulin-like growth factor 1 receptor,^[7–10]^ mammalian target of rapamycin,^[11,12]^ and poly adenosine diphosphate ribose polymerase 1,^[13,14]^ most target drugs have failed in their monotherapy phase II trials. By contrast, anti-angiogenesis tyrosine kinase inhibitors (aaTKIs), without gene-driven targets, have had some effect on sarcoma.^[15–22]^ Regorafenib, pazopanib, and apatinib have displayed activity in sporadic cases^[17–22]^ while cabozantinib^[23]^ appeared promising in a phase II trial, suggesting that aaTKIs are a promising treatment deserving of further investigation.

Anlotinib is a novel tyrosine kinase inhibitor that targets vascular endothelial growth factor receptor-2 and -3 and fibroblast growth factor receptor-1, -2, -3, and -4 with high affinity and broad antitumor activity against a variety of xenograft models.^[24,25]^ Unlike other aaTKIs, anlotinib has limited toxicity, with reactions in less than 20% of all patients in a phase II trial.^[26]^ Thus, this multicenter, two-armed phase Ib/II trial of Peking University People's Hospital Ewing Sarcoma trial-02 (PKUPH-EWS-02) investigated the recommended phase 2 dose and efficacy for anlotinib, vincristine, and 10-day irinotecan regimen for EWS progression upon the VDC/IE regimen.^[27]^ To move beyond tests of efficacy in both adult and pediatric patients and to determine the comparative effectiveness with real-world evidence, a follow-up study investigating the effects of these agents on quality of life (QoL) warranted exploration.

The quality-adjusted time without symptoms of disease progression or toxicity of treatment (Q-TWiST) analysis can be used to evaluate the overall effect of treatment interventions. Q-TWiST, categorized as a health index in the classification of benefit-risk methods described by Mt-Isa et al^[28]^ compares treatments by evaluating the quantity and quality of survival time using a single metric.^[29]^ Q-TWiST defines health states related to disease progression and toxicities, and it assigns QoL utility weights to each health state. By assessing QoL prospectively, this study aimed to compare the dynamic changes of QoL for patients receiving anlotinib and irinotecan in this trial. Moreover, we controlled for potential adverse events (AEs) by constructing mixed models and used Q-TWiST analysis to check the clinical benefits for these patients.

## 2 Methods

This study was approved by the Institutional Review Board of Peking University People's Hospital before commencement (2018PHD005-01) as well as Peking University Shougang Hospital. All participants provided written informed consent. All study procedures were performed in accordance with the International Council for Harmonization tripartite guideline on good clinical practice.^[30,31]^ One study amendment (protocol amendment version 3.0, June 29, 2018) was added to enable enrollment of children.

From March 5th, 2018 to October 5th, 2018, all patients with pathologically confirmed EWS, whose disease had progressed while on VDC/IE chemotherapy and had measurable lesions according to Response Evaluation Criteria in Solid Tumors version 1.1,^[32]^ were invited to join the study. Other inclusion criteria were (1) age ≥3 years; (2) adequate organ function; (3) Eastern Cooperative Oncology Group^[33]^ performance status 0 or 1; (4) estimated life expectancy ≥3 months; and (5) time elapsed from previous therapy ≥3 weeks for systemic therapy and ≥2 weeks for radiation therapy or major surgery. Patients who had poorly controlled hypertension, central nervous system metastasis, persistent clinically significant toxicities caused by previous therapy, or known active hepatitis B or C or human immunodeficiency virus were excluded from the study. Because we needed to analyze patients’ Q-TWiST time, all the intention-to-treat patients involved in phase II were included in this analysis.

The protocol treatment agents included anlotinib, irinotecan, and vincristine. The treatment regimen mainly consisted of a daily 90-minute intravenous administration of irinotecan d_1–5,__8–12_; 30-minute intravenous vincristine d_1,8;_ and oral anlotinib 2-week on, 1-week off,^[1–5,8–12]^ repeated every 21 days. For each patient, treatment was repeated until there was disease progression, unacceptable toxicity, or withdrawal of consent. Patients were enrolled into 2 arms according to age at enrollment: ≥16 years (“adult” patients) and <16 years (“pediatric” patients). We used the 30-item core European Organization for the Research and Treatment of Cancer Quality-of-Life Questionnaire (EORTC QLQ-C30) for adults (≥16 years) and the European Portuguese self-report version of the Pediatric Quality of Life Inventory Cancer Module (PedsQL 3.0 Cancer Module) for children and adolescents (<16 years) at baseline and at weeks 6, 12, 18, and 24 and after progression/off-treatment. (Full details of this trial can be found in https://www.clinicaltrials.gov/ct2/show/NCT03416517?term=NCT03416517&rank=1.)

Participants were invited to voluntarily complete the QoL questionnaires each time they visited the oncology clinics. For pediatric patients, the QoL questionnaires were completed separately by children/adolescents and their parents. EORTC QLQ-C30^[34]^ is one of the most widely used instruments for assessing health-related QoL in cancer patients; it contains multi-item scales: global health status (GHS); physical role; emotional, cognitive, and social functioning; fatigue; pain; and nausea plus vomiting. It also contains single-item measures of dyspnea, insomnia, anorexia, constipation, diarrhea, and financial impact. The PedsQL 3.0 Cancer Module^[35]^ is a disease-specific QoL instrument that measures the impact of symptoms and treatment on the QoL of pediatric patients with cancer; it consists of 27 items in 8 subscales: pain and hurt (2 items); nausea (5 items); procedural anxiety (3 items); treatment anxiety (3 items); worry (3 items); cognitive problems (5 items); perceived physical appearance (3 items); and communication (3 items). The PedsQL 3.0 Cancer Module is a multidimensional, easily comprehended, cancer-specific instrument designed for pediatric patients between the ages of 2 and 18 years, which is available in a self-report version designed for children/adolescents and a proxy-report version designed for guardians. All item scores of these two questionnaires are transformed to a range of 0 to 100, with higher scale scores representing a higher response level. The domain score was not calculated when more than two facets were missing from the domain. The repeated assessments were taken approximately 6 weeks apart to avoid high collinearity. The oncologists evaluated clinical efficacy for the patients every 6 weeks when they were receiving the study drug, during which time all questionnaires were filled out.

For the intention-to-treat population, AEs were recorded according to Common Terminology Criteria for Adverse Events version 4.03.^[36]^

The GHS/total scores from the EORTC QLQ-C30/ PedsQL 3.0 Cancer Module were used to perform the core analysis of QoL deterioration. We conducted analysis of mean age, distributions of gender, Eastern Cooperative Oncology Group performance status, and presence of metastasis between pediatric and adult patients. The basic characteristics or score levels of patients in the study groups were compared using Student *t* test for quantitative variables, and the chi-square test was used for categorical variables. We used Cox proportional hazard models to evaluate the hazard ratios of pediatric and adult patients for the time to the first deterioration, adjusted for covariate of interest. At each time point, the difference of mean scores of all domains of QoL between baseline and each time point were analyzed with pairwise comparison, either in independent-samples *t* test (Gaussian distribution) or Wilcoxon rank sum test (non-normal distribution) at a 5% level of significance. The risks of Grade 3/4 AEs in each trial were analyzed, and those with a risk ≈5% or higher were selected. The effect of each AE on concurrent GHS or total scores of pediatric patients was then analyzed by linear regression models to find factor(s) that affect QoL. The effects of this/these factor(s) on each functioning and symptom domains were further analyzed with linear regression models. All data were analyzed with R version 3.6.0 and SPSS for Windows software (ver. 20.0; SPSS Inc., Chicago, IL). All reported *P* values were 2-sided.

We applied the standard Q-TWiST methodology, wherein patient survival time was partitioned into three health states: (1) Time without symptoms or toxicities (TWiST): time from starting treatment to disease progression without ≥grade 3 AEs, which was defined as progression-free survival (PFS) time minus time with toxicity (TOX); (2) Toxicity: time with ≥grade 3 AEs after starting treatment and before disease progression; and (3) Relapse: time from disease progression to death, which was defined as overall survival (OS) minus PFS time. Patients who were alive or who were lost to follow-up were censored at the time of the last contact. For toxicity, the time spent with all-cause ≥grade 3 AEs before disease progression was summed for each patient, and a day with multiple events was only counted once. PFS was calculated as the time from initial treatment to progression, unacceptable toxicity, or death, whichever came first. OS was calculated as the interval from initial treatment to death. Survival curves that corresponded to toxicity, PFS, and OS were estimated with the Kaplan–Meier method. The restricted mean duration of each health state was derived from the area under the Kaplan–Meier curve. In this analysis, the mean Q-TWiST for both treatment arms was calculated together with 95% confidence intervals for the mean differences, in which variance was estimated by bootstrapping with 25,000 replications.^[37]^

## 3 Results

### 3.1 Patients’ characteristics

This exploratory study of QoL in EWS patients treated with combined anolotinib, irinotecan and vincristine included a sample of 35 individuals in accordance with the inclusion criteria. The patients’ baseline characteristics are presented in Table 1. Median follow-up time was 7.86 (interquartile range [IQR], 6.46–13.01) months. Twenty-three patients were assigned to the adult arm and 12 to the pediatric arm. The mean age of the 5 boys (41.7%) and 7 girls (58.3%) was 11.1 years (standard deviation = 2.77), while the mean age of the 18 male (78.3%) and 5 female (21.7%) “adults” was 27.5 years (standard deviation = 9.60). Distribution per age group was not uniform because of the small sample size (5–7, 16.7%; 8–12, 50.0%; 13–18, 41.7%). The distribution of patients was well balanced across treatment arms, except for gender distributions.

**Table 1:** Patients’ demographics by treatment group and completion rates of QoL questionnaires.

Demographic characteristics	Pediatric patients (N = 12)	Adult patients (N = 23)
Age		
Age (yr; mean ± SD)	11.08 ± 2.77	27.53 ± 9.60
Gender, N (%)		
Male	5 (41.67)	18 (78.26)
Female	7 (58.33)	5 (21.74)
ECOG performance status at enrollment, N (%)		
0	10 (100.00)	18 (78.26)
1	2 (16.7)	5 (21.74)
Presence of metastasis, N (%)		
No (locally advanced)	2 (16.67)	1 (4.35)
Yes	10 (83.33)	22 (95.65)
Primary tumor location, N (%)		
Extremities	5 (41.67)	9 (39.13)
Axial skeleton	3 (25.00)	6 (26.09)
Others^∗^	4 (33.33)	8 (34.78)
Sites of lesions, N (%)		
Lung only	4 (33.33)	11 (47.83)
Bone only	1 (8.33)	0 (0.00)
Lung and bone or viscera	7 (58.33)	12 (52.17)
Time interval from diagnosis to enrollment, N (%)		
≤24 mo	12 (100.00)	21 (91.30)
>24 mo	0 (0.00)	2 (8.70)
Lines of previous chemotherapy, N (%)		
1	8 (66.67)	15 (65.22)
≥2	4 (33.33)	8 (34.78)
Previous radiotherapy, N (%)		
No	9 (75.00)	12 (52.17)
Yes	3 (25.00)	11 (47.82)
Combined with EWS-FLI1 translocation,^†^ N (%)		
No	1 (8.33)	1 (4.35)
Yes	9 (75.00)	11 (47.83)
Unknown	2 (16.67)	11 (47.83)
Lactate dehydrogenase > ULN, N (%)		
No	10 (83.33)	14 (60.87)
Yes	2 (16.67)	9 (39.13)
UGT1A1∗1 mutation,^‡^ N (%)		
Wild type	7 (58.33)	12 (52.17)
Homozygous mutation	0 (0.00)	0 (0.00)
Heterozygous mutation	5 (41.67)	5 (21.74)
Unknown	0 (0.00)	6 (26.09)
UGT1A1∗28 mutation, N (%)		
Wild type	11 (91.67)	12 (52.17)
Homozygous mutation	0 (0.00)	0 (0.00)
Heterozygous mutation	1 (8.34)	5 (21.74)
Unknown	0 (0.00)	6 (26.09)
Completion rates of QoL questionnaires,^§^ N (%)		
Baseline	12/12 (100.00)	23/23 (100.00)
Week 6	12/12 (100.00)	22/23 (95.65)
Week 12	11/11 (100.00)	17/17 (100.00)
Week 18	7/7 (100.00)	13/13 (100.00)
Week 24	2/2 (100.00)	7/7 (100.00)
Off-treatment/progression	7/10 (70.00)	14/23 (60.87)

∗ Others included intraperitoneal infiltration.

† Translocation of EWSR1 on chromosome 22 to chromosome 11 occurs in 85% of Ewing sarcoma cases, forming the fusion protein product EWS-FLI1.

‡ The general status, including basic functions of the major organs and the UGT1A1 (key enzyme in the glucuronidation of SN38 in the liver) genotype, are two major factors used to assess the risk of irinotecan-induced diarrhea. However, although agreement has been reached in the predictive value of the UGT1A1 genotype in colorectal cancer, the detection of the UGT1A1 genotype is not typically recommended in protracted schedules in pediatric patients. Unfortunately, no other genetic markers have been found for this group.

§ Completion rates of QoL questionnaires means the ratio of patients who completed the health-related quality of life questionnaires to all those who stayed in the trial at various times. ECOG = Eastern Cooperative Oncology Group, EWS = Ewing sarcoma, EWSR1 = Ewing sarcoma breakpoint region 1, QoL = quality of life, SD = standard deviation, UGT1A1 = uridine diphosphate glucuronic acid transferase 1A1, ULN = upper limit of normal.

Completion rates for the QoL questionnaires in the populations are also listed in Table 1. Compliance with QoL assessment was excellent; 100% of patients completed the questionnaires at baseline. Compliance rates slowly decreased over time, with the lowest rate reported at off-treatment/progression (70.0% and 60.9% for pediatric and adult arms, respectively). No statistically significant differences in compliance rates between the 2 arms were observed at any time. The median number of completed questionnaires was 4 for the pediatric group and 5 for the adult group. The mean and median QoL scores at baseline for the total scales were comparable between the 2 age groups. Compared with other studies, the compliance rates in our trial were acceptable.^[38]^

### 3.2 Dynamic changes of QoL after treatment

**Figure 1:**
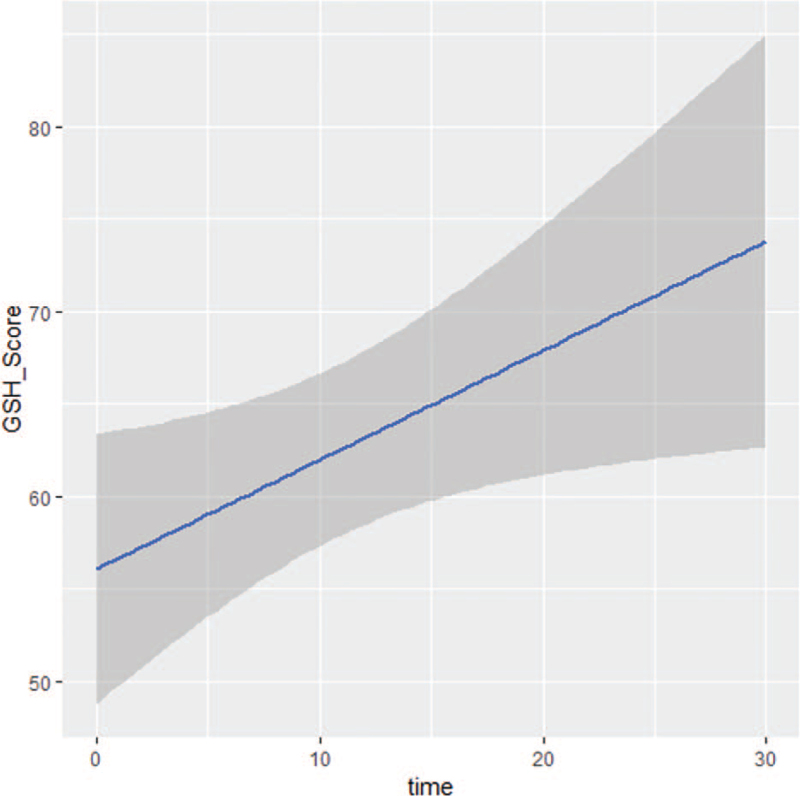
Dynamic changes of adults’ GHS scores according to EORTC QLQ-C30 over time. EORTC QLQ-C30 = 30-item core European Organization for the Research and Treatment of Cancer Quality-of-Life Questionnaire, GHS = global health status.

**Figure 2:**
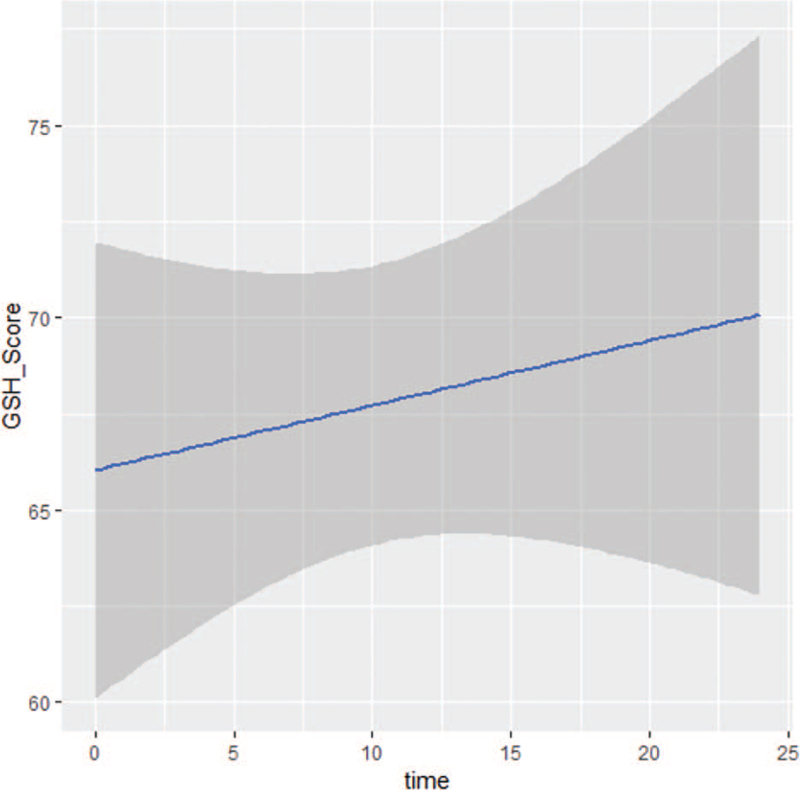
Dynamic changes of children's general scores (GHS) according to PedsQL3.0 Generic Core Scales over time. GHS = global health status.

Table 2 depicts the global and functioning scales in the 23 adult patients. The GHS values increased modestly from baseline (58.3 ± 25.5) to week 24 (73.8 ± 13.1), but the difference was not statistically significant (*P* = .14). Whether the trend to improved GHS values was due to decreased tumor burden is a possibility but is unproven. QoL declined by about one-half with disease progression. Among the other scales of the EORTC QLQ-C30, there were both statistically and clinically significant differences in scores between baseline and during treatment for diarrhea, loss of appetite, fatigue, and financial difficulties. QoL decreased markedly with disease progression. For repeated assessments within individual subjects, the dynamic changes of QoL were investigated using a linear model, which are presented in Figures 1 and 2.

**Table 2:** Analyses of EORTC QLQ-C30 global and functioning scales for adult patients (N = 23).

EORTC QLQ-C30	Baseline	Week 6	Week 12	Week 18	Week 24	Off-treatment	Progression
N	23	22	17	13	7	9	4
Global health status/QoL (0–100)							
Mean ± SD	58.33 ± 25.5	57.95 ± 21.89	64.22 ± 24.25	67.95 ± 21.2	73.81 ± 13.11	77.08 ± 11.57	35.42 ± 25.8
Change from baseline, mean ± SD		−0.76 ± 24.24	4.90 ± 21.86	−0.69 ± 17.59	3.57 ± 15.99	4.76 ± 10.20	−16.67 ± 20.84
*P* value (compared with baseline, *t* test)		.96	.47	.26	.14	.14	.11
Physical functioning (0–100)							
Mean ± SD	77.39 ± 25.32	73.03 ± 21.92	74.51 ± 22.76	83.08 ± 7	80.00 ± 6.67	75.83 ± 14.23	61.67 ± 41.59
Change from baseline, mean ± SD		−3.94 ± 16.06	−5.49 ± 11.95	−2.22 ± 10.74	−4.76 ± 12.52	−9.52 ± 8.98	−20.00 ± 26.67
*P* value (compared with baseline, *t* test)		.54	.71	.44	.79	.79	.31
Role functioning (0–100)							
Mean ± SD	69.57 ± 30.01	62.12 ± 29.63	68.63 ± 26.27	67.95 ± 24.96	73.81 ± 13.11	66.67 ± 19.92	62.50 ± 43.83
Change from baseline, mean ± SD		−7.94 ± 23.36	−3.92 ± 21.80	−5.56 ± 23.15	4.76 ± 19.73	−4.76 ± 6.80	−16.67 ± 16.67
*P* value (compared with baseline, *t* test)		.41	.92	.87	.72	.72	.69
Emotional functioning (0−100)							
Mean ± SD	77.90 ± 21.41	75.76 ± 23.98	72.55 ± 24.78	81.41 ± 25.49	84.52 ± 19.5	78.13 ± 16.63	62.50 ± 35.03
Change from baseline, mean ± SD		−1.14 ± 16.60	−9.31 ± 14.13	−2.78 ± 21.76	−1.19 ± 15.99	2.38 ± 21.09	−25.00 ± 16.67
*P* value (compared with baseline, *t* test)		.75	.47	.66	.47	.47	.24
Cognitive functioning (0–100)							
Mean ± SD	86.23 ± 17.15	84.85 ± 14.46	81.37 ± 20.31	82.05 ± 24.96	85.71 ± 11.5	81.25 ± 13.91	70.83 ± 47.87
Change from baseline, mean ± SD		−1.59 ± 14.81	−5.88 ± 18.68	−8.33 ± 19.44	−2.38 ± 16.33	−9.52 ± 8.16	−20.83 ± 31.25
*P* value (compared with baseline, *t* test)		.77	.42	.56	.94	.94	.23
Social functioning (0–100)							
Mean ± SD	61.59 ± 29.06	54.55 ± 24.22	59.80 ± 25.72	64.10 ± 19.06	50.00 ± 16.67	58.33 ± 29.55	41.67 ± 50
Change from baseline, mean ± SD		−6.82 ± 22.59	−5.88 ± 17.99	−2.78 ± 11.57	−16.67 ± 9.53	0.00 ± 14.29	−37.50 ± 37.50
*P* value (compared with baseline, *t* test)		.38	.84	.78	.33	.33	.27
Symptom domains							
Fatigue, mean ± SD	34.30 ± 23.90	36.36 ± 20.00	32.03 ± 21.83	30.77 ± 21.35	26.98 ± 8.74	20.00 ± 19.47	55.56 ± 27.22
Nausea and vomiting, mean ± SD	12.32 ± 18.95	15.91 ± 18.88	15.67 ± 25.69	15.38 ± 28.43	4.76 ± 8.13	6.25 ± 12.40	25.00 ± 21.52
Pain, mean ± SD	20.29 ± 27.04	24.24 ± 23.42	13.73 ± 23.00	14.10 ± 14.98	16.76 ± 13.61	12.50 ± 23.15	54.17 ± 34.36
Dyspnea, mean ± SD	13.04 ± 21.88	6.06 ± 13.16	7.84 ± 14.57	7.69 ± 14.62	4.76 ± 12.60	12.50 ± 17.25	33.33 ± 47.14
Insomnia, mean ± SD	17.39 ± 22.18	13.64 ± 19.68	15.69 ± 17.15	7.69 ± 19.97	14.29 ± 17.82	12.50 ± 17.25	16.67 ± 19.25
Appetite loss, mean ± SD	21.74 ± 21.58	27.26 ± 24.42	23.53 ± 19.60	30.77 ± 16.45	23.81 ± 16.27	8.33 ± 15.43	33.33 ± 27.22
Constipation, mean ± SD	13.04 ± 24.08	9.09 ± 18.35	5.88 ± 13.10	10.26 ± 21.01	4.76 ± 12.60	12.50 ± 17.25	25.00 ± 31.91
Diarrhea, mean ± SD	7.25 ± 14.06	39.39 ± 24.42	39.22 ± 29.43	35.90 ± 25.32	23.81 ± 16.27	12.50 ± 17.25	50.00 ± 33.33
Financial difficulties, mean ± SD	57.97 ± 39.21	60.61 ± 31.93	52.94 ± 31.31	51.28 ± 32.25	52.38 ± 32.53	50.00 ± 39.84	66.67 ± 38.49

EORTC QLQ-C30 = 30-item core European Organization for the Research and Treatment of Cancer Quality-of-Life Questionnaire, QoL = quality of life, SD = standard deviation.

**Figure 3:**
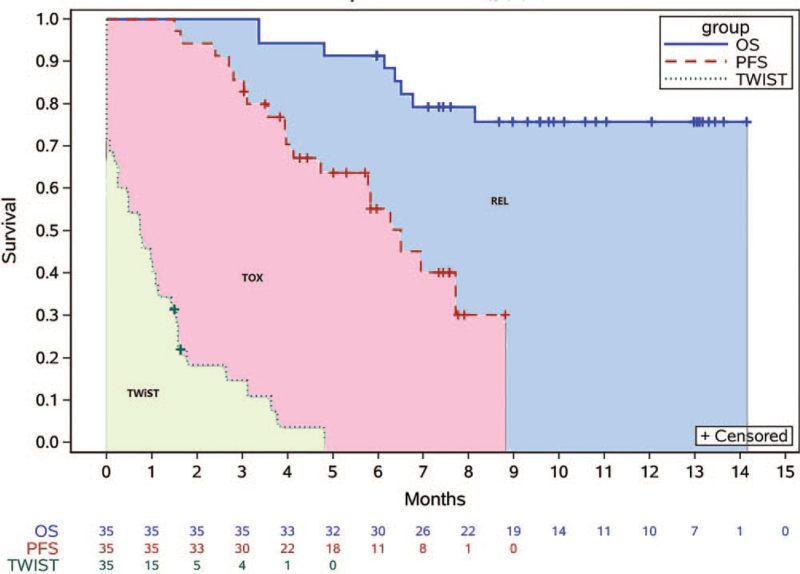
Dynamic changes of adults’ functioning scales for adults according to EORTC QLQ-C30 over time, which included physical, role, emotional, cognitive, and social functioning. EORTC QLQ-C30 = 30-item core European Organization for the Research and Treatment of Cancer Quality-of-Life Questionnaire, OS = overall survival, PFS = progression-free survival, REL = relapse, TOX = toxicity, TWiST = time without symptoms or toxicities.

**Figure 4:**
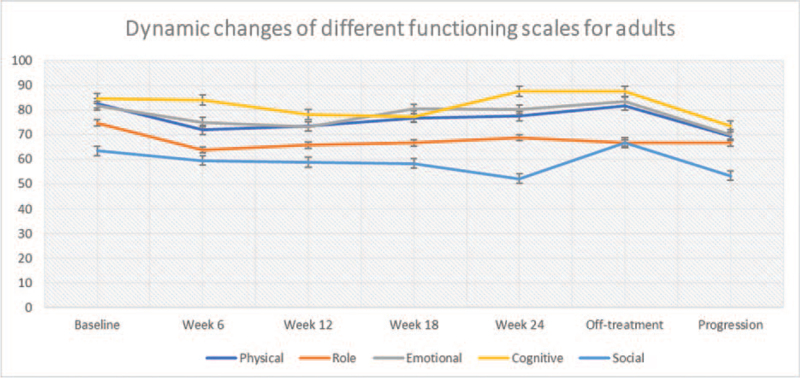
Dynamic changes of children's scale descriptives of QoL according to EORTC QLQ-C30 over time, which included pain and hurt (2 items), nausea (5 items), procedural anxiety (3 items), treatment anxiety (3 items), worry (3 items), cognitive problems (5 items), perceived physical appearance (3 items), and communication (3 items). EORTC QLQ-C30 = 30-item core European Organization for the Research and Treatment of Cancer Quality-of-Life Questionnaire, QoL = quality of life.

Table 3 presents the PedsQL3.0 generic core scales for child self-reports and parent proxy reports during the trial. Total scores for both reports did not change significantly from baseline to week 24 of treatment, and there was no difference in values between the pediatric and adult treatment arms. With disease progression, however, the scores for pediatric patients, as for “adult” patients, declined. In comparison of scores between child self-reporting and parent proxy reporting, correlation was good (>0.70) for all variables, except for “worry” (0.64). We analyzed these items’ dynamic changes with linear models constructed to explore the determinants of QoL, as illustrated in Figures 3 and 4.

**Table 3:** Scale descriptors and test-retest reliability analysis for PedsQL3.0 Generic Core Scales child self- and parent proxy-report.

Scale	Baseline (mean, SD)	Week 6 (mean, SD)	Week 12 (mean, SD)	Week 18 (mean, SD)	Week 24 (mean, SD)	Off-treatment (mean, SD)	Progression (mean, SD)	Intraclass correlation efficient, ICC^∗^
N	12	12	11	7	2	5	2	
Child self-report								
Total score	64.62 ± 13.72	67.79 ± 13.54	72.59 ± 10.39	71.90 ± 10.51	75.93 ± 3.93	66.11 ± 6.95	41.20 ± 12.44	0.908
Change from baseline, mean ± SD		3.16 ± 9.77	8.83 ± 16.10	3.97 ± 13.80	18.52 ± 5.24	−1.49 ± 2.59	−23.42 ± 7.37	
*P* value (compared with baseline, *t* test)		.286	.10	.475	.126	.384	.898	
Pain and hurt	68.75 ± 23.54	82.29 ± 18.04	78.41 ± 19.44	78.57 ± 18.70	62.50 ± 17.68	52.50 ± 18.54	N/A	0.901
Nausea	52.50 ± 10.77	46.25 ± 18.60	61.82 ± 13.47	63.57 ± 14.06	60.00 ± 14.14	58.00 ± 10.37	42.50 ± 24.75	0.864
Procedural anxiety	63.89 ± 32.44	65.97 ± 25.24	60.61 ± 30.30	60.71 ± 13.36	75.00 ± 0	60.00 ± 18.07	70.83 ± 29.46	0.750
Treatment anxiety	68.75 ± 27.55	77.08 ± 25.65	81.06 ± 18.29	78.57 ± 15.85	87.50 ± 17.68	68.33 ± 12.64	50.00 ± 23.57	0.731
Worry	51.39 ± 30.33	62.50 ± 28.32	71.97 ± 15.49	71.43 ± 20.89	87.50 ± 17.68	63.33 ± 12.64	40.00 ± 14.14	0.636
Cognitive problems	72.71 ± 16.01	72.92 ± 11.96	79.77 ± 12.57	72.26 ± 10.79	85.00 ± 14.14	77.00 ± 13.96	33.33 ± 47.14	0.722
Perceived physical appearance	65.28 ± 16.98	76.39 ± 15.42	74.24 ± 16.01	75.00 ± 12.73	75.00 ± 0	75.00 ± 16.67	45.83 ± 29.46	0.745
Communication	77.08 ± 18.16	74.31 ± 22.04	77.27 ± 18.29	82.14 ± 15.54	75.00 ± 0	68.33 ± 24.58	41.20 ± 12.44	0.705
Guardian report								
Total score	65.09 ± 12.28	68.02 ± 12.24	70.24 ± 12.56	71.97 ± 7.50	64.81 ± 6.55	65.56 ± 5.87	42.28 ± 16.58	
Change from baseline, mean ± SD		2.93 ± 8.89	5.30 ± 17.12	3.06 ± 11.91	3.70 ± 9.17	0.47 ± 5.25	22.81 ± 11.72	
*P* value (compared with baseline, *t* test)		.279	.329	.557	.670	.346	.610	
Pain and hurt	73.96 ± 23.51	73.96 ± 16.39	76.14 ± 23.35	77.08 ± 16.61	75.00 ± 35.36	55.00 ± 20.92	12.50 ± 17.68	
Nausea	54.17 ± 7.93	51.25 ± 20.79	57.73 ± 15.06	62.50 ± 14.05	50.00 ± 0	58.00 ± 11.51	35.00 ± 28.28	
Procedural anxiety	65.28 ± 27.26	65.97 ± 23.69	65.91 ± 23.70	62.50 ± 18.82	75.00 ± 11.79	65.00 ± 13.69	29.17 ± 29.46	
Treatment anxiety	71.53 ± 27.86	79.17 ± 21.76	78.03 ± 21.50	79.17 ± 17.28	70.83 ± 5.89	66.67 ± 11.79	29.17 ± 41.25	
Worry	44.44 ± 31.45	56.25 ± 36.61	58.33 ± 27.64	69.44 ± 18.00	54.17 ± 5.89	53.33 ± 26.74	25.00 ± 35.36	
Cognitive problems	73.89 ± 14.50	73.33 ± 12.27	80.15 ± 13.43	79.44 ± 16.28	75.00 ± 0	78.00 ± 13.51	66.67 ± 11.79	
Perceived physical appearance	66.67 ± 15.49	76.39 ± 17.35	72.73 ± 18.67	73.61 ± 16.17	54.17 ± 5.89	71.67 ± 16.24	41.67 ± 0.00	
Communication	75.69 ± 17.21	77.08 ± 19.18	77.27 ± 27.15	77.78 ± 18.76	70.83 ± 5.89	70.00 ± 24.01	79.17 ± 5.89	

∗ The uniformity and correlation analysis between child self-report and guardian report QoL. ICC ≤0.7 indicates good uniformity and correlation. ICC = intraclass correlation efficient, N/A = not available, PedsQL 3.0 = European Portuguese self-report version of the Pediatric Quality of Life Inventory, QoL = quality of life, SD = standard deviation.

### 3.3 Grade 3/4 AEs influenced QoL

In the trial, the most common AEs were leukopenia (16.6%), anemia (12.7%), neutropenia (12.1%), and diarrhea (4.93%). We compared QoL in patients during periods of grade 3/4 AE associated with neutrophil decrease, white blood cell decrease, anemia, and diarrhea. For adult patients (Table 4), leukopenia was associated with diminished GHS (*P* = .015), neutropenia with decreased physical functioning (*P* = .046), and diarrhea with diminished cognitive functioning (*P* = .042). For pediatric patients (Table 5), severe diarrhea was associated with significantly lower descriptive scales of “pain and hurt” (*P* = .013) and “perceived physical appearance” (*P* = .011), while leukopenia was associated with lower “procedural anxiety” scores (*P* = .014). Neither leukopenia nor diarrhea was associated with other descriptive scales or symptoms domains (all *P* > .05).

**Table 4:** The relationship between grade 3/4 AEs (incidence ≥5%) and QoL.

Grade 3/4 AEs	NE decrease		WBC decrease		Anemia		Diarrhea	
	Yes	No	Yes	No	Yes	No	Yes	No
Global health status (0–100)								
Mean ± SD	66.35 ± 22.42	61.35 ± 23.57	58.72 ± 23.78	70.97 ± 20.05	51.12 ± 24.26	62.22 ± 24.68	61.90 ± 32.93	61.39 ± 24.17
*P* value (*t* test)	.350		.015		.828		.367	
Physical functioning (0–100)								
Mean ± SD	69.23 ± 26.20	78.94 ± 18.56	75.05 ± 22.18	76.88 ± 20.91	63.81 ± 30.27	75.56 ± 23.07	72.38 ± 33.21	74.89 ± 23.01
*P* value (*t* test)	.046		.697		.196		.494	
Role functioning (0–100)								
Mean ± SD	62.82 ± 27.61	68.84 ± 26.49	66.67 ± 26.87	67.45 ± 26.96	59.52 ± 34.50	66.30 ± 27.82	73.81 ± 35.82	65.19 ± 27.67
*P* value (*t* test)	.332		.895		.317		.492	
Emotional functioning (0–100)								
Mean ± SD	79.49 ± 22.01	75.85 ± 23.40	77.96 ± 23.03	76.30 ± 23.11	65.48 ± 27.82	76.02 ± 25.04	79.76 ± 29.99	74.91 ± 25.00
*P* value (*t* test)	.494		.744		.863		.577	
Cognitive functioning (0–100)								
Mean ± SD	83.97 ± 14.51	83.09 ± 20.91	81.18 ± 17.07	84.38 ± 20.33	83.33 ± 13.61	81.48 ± 23.09	76.19 ± 37.09	82.04 ± 21.23
*P* value (*t* test)	.834		.453		.370		.042	
Social functioning (0–100)								
Mean ± SD	57.69 ± 25.49	58.70 ± 26.91	62.37 ± 23.56	56.51 ± 27.64	54.76 ± 24.93	57.41 ± 27.72	69.05 ± 36.55	56.30 ± 26.63
*P* value (*t* test)	.870		.313		.649		.450	

AEs = adverse events, NE = neutrophils, QoL = quality of life, SD = standard deviation, WBC = while blood cells.

**Table 5:** The relationship with Grade 3/4 AEs (incidence ≥5%) with QoL of pediatric patients.

Grade 3/4 AEs	NE decrease		WBC decrease		Anemia		Diarrhea	
	Yes	No	Yes	No	Yes	No	Yes	No
Total (0–100)								
Mean ± SD	66.57 ± 12.94	68.12 ± 12.93	66.66 ± 9.64	68.02 ± 14.46	73.23 ± 6.96	66.89 ± 13.39	51.39 ± 26.84	68.42 ± 12.05
*P* value (*t* test)	.676		.716		.170		.086	
Pain and hurt (0–100)								
Mean ± SD	71.86 ± 28.35	69.53 ± 23.32	70.39 ± 21.73	70.45 ± 27.21	80.36 ± 20.23	69.32 ± 25.78	43.75 ± 61.87	71.94 ± 23.46
*P* value (*t* test)	.747		.994		.481		.013	
Nausea (0–100)								
Mean ± SD	51.00 ± 11.54	57.19 ± 16.89	55.79 ± 11.34	54.24 ± 17.24	64.29 ± 10.97	53.52 ± 15.50	35.00 ± 14.14	55.82 ± 14.94
*P* value (*t* test)	.156		.728		.804		.967	
Procedural anxiety (0–100)								
Mean ± SD	56.67 ± 24.87	67.97 ± 24.33	52.63 ± 24.22	69.95 ± 23.38	53.57 ± 28.41	65.15 ± 24.59	91.67 ± 0.00	62.42 ± 25.01
*P* value (*t* test)	.112		.014		.675		.070	
Treatment anxiety (0–100)								
Mean ± SD	76.67 ± 21.22	73.18 ± 22.67	75.44 ± 20.87	73.99 ± 22.89	84.52 ± 14.77	73.11 ± 22.87	75.00 ± 11.79	74.66 ± 22.56
*P* value (*t* test)	.583		.822		.577		.516	
Worry (0–100)								
Mean ± SD	69.17 ± 25.38	57.55 ± 25.69	70.18 ± 21.57	57.32 ± 27.38	83.33 ± 15.96	59.85 ± 24.86	41.67 ± 35.36	63.95 ± 24.67
*P* value (*t* test)	.117		.09		.374		.582	
Cognitive problems (0–100)								
Mean ± SD	71.88 ± 14.98	75.26 ± 14.49	72.63 ± 10.98	74.72 ± 16.48	76.19 ± 9.74	73.35 ± 15.42	52.50 ± 31.82	74.71 ± 13.62
*P* value (*t* test)	.422		.624		.216		.055	
Perceived physical appearance (0–100)								
Mean ± SD	71.67 ± 22.36	70.83 ± 15.12	72.37 ± 13.62	70.45 ± 20.32	76.90 ± 17.63	70.45 ± 18.37	29.17 ± 41.25	72.96 ± 15.26
*P* value (*t* test)	.873		.716		.939		.011	
Communication (0–100)								
Mean ± SD	72.08 ± 24.52	76.04 ± 16.50	68.42 ± 18.34	78.03 ± 20.07	72.62 ± 15.75	75.38 ± 20.41	50.00 ± 35.36	76.02 ± 18.76
*P* value (*t* test)	.489		.093		.233		.209	

AEs = adverse events, NE = neutrophils, QoL = quality of life, SD = standard deviation, WBC = while blood cells.

### 3.4 Duration of the health states

**Figure 5:**
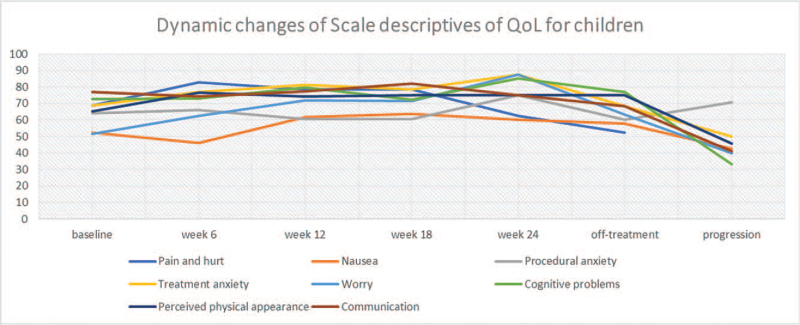
All patients’ Q-TWiST over time. Crosses indicate censoring. (1) EWiST: time from starting treatment to disease progression without ≥grade 3 AEs, which was defined as PFS time minus time with TOX; (2) TOX: time with ≥grade 3 AEs after starting treatment and before disease progression; (3) REL: time from disease progression to death, which was defined as OS minus PFS time. AEs = adverse events, PFS = progression-free survival, OS = overall survival, Q-TWiST = Quality-adjusted Time Without Symptoms of disease progression or Toxicity of treatment, REL = relapse, TOX = toxicity, TWiST = time without symptoms or toxicities.

Because of small sample size, partitioned survival plots of both populations, based on PKUPH-EWS02, were determined (Fig. 5). The area between the curves illustrates the time in each of the three health states in the Q-TWiST calculation. Parameters of the time periods are listed in Table 6. The primary results revealed that patients receiving the combination therapy of anlotinib, irinotecan, and vincristine had a median TWiST of 0.73 (IQR, 0–1.57) months; nevertheless, the median time with toxicity was 3.9 (IQR, 2.3–6.1) months. Because most patients reached complete response or partial response during the trial, we recommended local therapy (radiotherapy or surgery) for residual tumor lesions; if patients had multiple pulmonary metastasis after complete response or nearly complete response, we recommended whole-lung irradiation to prevent tumor relapse. Thus, most patients left the trial not because of progression of disease but to receive local therapy. The curve for PFS is the time for off-treatment (because of local therapy) or progression. The time after progression is pending, as the focus of this study is the QoL during treatment.

**Table 6:** Restricted mean durations of health states.

Health state	N = 35
Mean PFS, month (95% CI)	5.73 (4.99–11.37)
Median PFS, month (IQR)	6.50 (2.7–6.93)
Mean OS, month (95% CI)	7.56 (N/A)
Median OS, month (IQR)	N/A
Mean TOX, month (95% CI)	4.0 (3.2, 4.8)
Median TOX, month (IQR)	3.9 (2.3, 6.1)
Mean Q-TWiST, month (95% CI)	1.14 (0.69–1.59)
Median Q-TWiST, month (IQR)	0.73 (0–1.57)

CI = confidence interval, IQR = interquartile range, N/A = not available, OS = overall survival, PFS = progression-free survival, Q-TWiST = Quality-adjusted Time Without Symptoms of Disease Progression or Toxicity of Treatment, TOX = toxicity, TWiST = time without symptoms or toxicities.

## 4 Discussion

EWS family tumors, heretofore referred to as Ewing's sarcoma, are found primarily in adolescents and young adults, with peak occurrence between 5 and 20 years old;^[3]^ they are genetically characterized by chromosomal translocation involving the Ewing sarcoma breakpoint region 1 gene.^[39]^ As a novel regimen for the treatment of metastatic Ewing's sarcoma, the combination of anlotinib, vincristine, and 5d∗2 protracted regimen of irinotecan has been shown to significantly shrink tumors and substantially prolong patient survival without adding treatment-related toxicity in patients whose disease had progressed on traditional first-line chemotherapy of VDC/IE or vincristine, ifosfamide, doxorubicin, and etoposide.^[27]^ This is the first QoL analysis to quantify the benefit-risk of this therapeutic strategy by concurrently evaluating dynamic changes of QoL, survival time, disease progression, and safety profile throughout the clinical trial PKUPH-EWS-02.

We had tried to compare the QoL for second-line treatment for EWS with previous publications.^[40,41]^ However, due to the rare disease with small sample sizes transferred into advanced stages, there had been few publications for this group of patients with diverse questionnaires.^[40,41]^ At the same time together with other sacomas, in the most recent QoL analysis of the phase 3 pazopanib for metastatic soft-tissue sarcoma trial, no statistically or clinically significant differences of EORTC QLQ-C30 GHS between the pazopanib arm and placebo arms were present at any time point.^[42]^ However, the toxicity profile of pazopanib was reflected in patients’ self-reported symptoms (fatigue, nausea/vomiting, appetite loss, and diarrhea), and QoL scores declined over time in both arms. As with previous reports, slightly lower values or equivalent fluctuations of QoL were present in most patients receiving aaTKIs, including cabozantinib,^[43]^ sorafenib,^[44–47]^ and sunitinib.^[45]^ By using a linear regression model in this study, we found a trend toward improving QoL during treatment, more so for “adults” than for younger patients. Thus, on the basis of patients’ self-reported QoL, this combination therapy seemed to be adoptable. The analysis of pediatric patients’ QoL was more complex. For example, the questionnaires included one part for the children/adolescents and the other for their guardians. However, the scores for both cohorts in the PedsQL Cancer Module subscales were well correlated. Moreover, the 6-week interval between interviews diminished the probability of systemic alterations in the patients’ clinical conditions. For all patients, descriptive scales and symptom scales did not decline over time, except for disease progression.

Significantly worse outcomes in the adult arm were observed for 3 symptoms scales: diarrhea, appetite loss, and fatigue. This result is in line with the toxicity profile of irinotecan and angiogenesis inhibitors. The most frequently cited side effects of this combination treatment are leukocytosis, anemia, agranulocytosis, and diarrhea. In this study, the most common grade 3/4 adverse effects, leukocytosis and diarrhea, were the main factors that significantly impaired GHS in adults, and they were associated with procedural anxiety, pain and hurt, and perceived physical appearance in children. Anemia, recorded 160/1258 (12.7%) times, was one of the most frequent AEs, but it did not influence QoL, either in adults or pediatric patients. Nonetheless, chronic anemia should be heeded in heavily treated Ewing patients, and intermittent blood infusion may be required.

We noticed a large portion of area under curve of TOX rather than TWiST in our study, which was also in accordance with other trials for advanced solid tumors.^[48–50]^ However, the most significant part of these curves is how could we improve the Q-TWiST with improvement of drug or methods over time. However, because of insufficient QoL data on advanced Ewing's sarcoma and other trials, no comparison could be made to identify the advantages and disadvantages of this treatment.

Our exploratory analyses have strengths and limitations. A strength was the systematic and prospective collection of data with relatively good compliance. Although the number of questionnaires filled out decreased over time, sufficient data were accrued to permit hypothesis-generating analyses. Limitations were as follows: First, the follow-up time was not long enough to determine whether the trial yielded a survival benefit. The relapse time and toxicity time should be checked again when PFS and OS data collection is complete. Second, numerous censorings, because of local therapy after 12 weeks of study drug administration, resulting in disappearance of lesions or decreasing stage of sarcoma, caused differences of QoL between off-treatment and progression. For the benefit of patients, this processing mode was acceptable in cases of secondary resistance of drugs, while for the primary end point of this 1B trial, we obtained a 12-week objective response rate and recommended phase 2 dose, which was also reasonable. For survival data, however, this was not satisfactory because of intervention with local therapy. Third, the adults and children/adolescents completed different questionnaires of QoL. Thus, the results were heterogeneous, making the analysis difficult and complicated. Finally, this IB trial is limited by the relatively small sample size and absence of a control group. Further investigation is expected to compensate for this defect.

## 5 Conclusions

In this quality-of-life and Q-TWiST analysis of EWS patients treated with anlotinib, irinotecan, and vincristine, PFS was not accompanied by diminished QoL. Patients’ self-reported symptoms did not translate into significantly worse overall scores during treatment. Thus, the combination therapy with anlotinib, irinotecan, and vincristine for advanced EWS has an acceptable effect on health-related QoL.

## Acknowledgments

The authors thank all the patients and their families for participating in this clinical trial. The authors also thank Medlink (the third party for data analysis) for interpretation of all the clinical data and statistical analysis of this trial.

## Author contributions

**Conceptualization:** Lu Xie, Jin Gu, Wei Guo.

**Data curation:** Jie Xu, Xin Sun, Yi Huang, Jin Gu.

**Formal analysis:** Xin Sun, Yi Huang.

**Funding acquisition:** Wei Guo.

**Investigation:** Kunkun Sun, Rongli Yang.

**Methodology:** Kunkun Sun, Jie Xu, Rongli Yang.

**Project administration:** Kunkun Sun, Xin Sun, Tingting Ren, Rongli Yang, Fan Yang.

**Resources:** Tingting Ren, Xiaodong Tang, Fan Yang.

**Software:** Sen Dong, Tingting Ren.

**Supervision:** Xiaodong Tang, Wei Guo.

**Validation:** Jie Xu, Xiaodong Tang, Wei Guo.

**Visualization:** Wei Guo.

**Writing – original draft:** Sen Dong, Kunkun Sun.

**Writing – review & editing:** Lu Xie, Wei Guo.
